# Publishing in learned journals

**Published:** 2010-11

**Authors:** S.N. Arseculeratne

**Affiliations:** Department of Microbiology, Faculty of Medicine, University of Peradeniya, Peradeniya, Sri Lanka chubby@sltnet.lk

Sir,

Apropos to the Editorial recently published on biomedical journals in India^1^.

Journals of course are of great importance in the advancement of science and I recall Charles Singer’s view (in his short history of modern science) of the necessity to have scientific findings exposed for verification or falsification in this process of the growth and advancement of science. One factor that inhibited the growth and emergence of modern science in pre-modern south Asia was the virtual absence of scientific societies and mechanisms for airing scientific findings^2^. On the other hand, researchers might, for whatever reason, be disinclined to publicize their findings or communicate with learned societies.

The fact, which was pointed out, that articles from South Asia do not appear, as often as they should, in western journals that are regarded as high Impact Factor (IF) journals is not always due to inferior quality. There is evidence of bad refereeing and discrimination by the western journals, for which I have much evidence. Here are some:-

- A leading medical journal in the UK declined to publish a letter from us that showed, substantiating a first report from Scandinavian workers in the same journal, that intradermal typhoid vaccination was effective especially in inducing cell-mediated immunity^3^. That same journal published a letter from western authors, the only content of which was the popular cartoon of a face with a smile that the authors said they found in one of their microscope-fields. I did not know that this ‘scientific’ journal was competing with the humour magazine “*Punch*”. The letter itself was not published elsewhere later but the full article was^3^.
- A US reviewer of one of our submissions to a prestigious mycology journal in the US made negative comments in declining acceptance that indicated that he was not aware of the difference between an antiseptic and an antibiotic.
- Another article that dealt with a new identification of a phenomenon in aflatoxin accumulation in foodstuffs, a very important topic in food-safety, was commented on by a reviewer also in the US as a technical error. The article was subsequently published unchanged in a British journal^4^.
- An important article, a recommended reading in a major textbook on Medicine in the UK, was commented on by a leading UK journal as dealing with a very minor aspect. The same article was published by the same Journal^5^on the very positive recommendation of the second reviewer.
- Since players of the IF game are mainly ‘journalists’ of the so-called high IF western journals, it becomes a vicious tautology, selective refereeing → selective indexing → citation indices identifying high IF journals → selective refereeing.

Only articles published in the so-called prestigious western journals are considered as containing “evidence”. Important findings by Asian authors are excluded as they do not find publication space in these so-called “high impact” (IF) factor journals. This has led to the emergence of a new disease called “Impactitis”^6^,^7^.

The point is that not all important, valid articles from South Asia find publication space in so-called prestigious journals of the West. This leads to a very important implication, that of Evidence-based Medicine, which is really Evidence-based Medicine from the West. The only remedy is of course to upgrade the quality of our regional journals and it was, therefore, a good idea of WHO/SEARO to have initiated a Regional Index Medicus to include these articles from Asian authors that are ignored by western journals. The second emerging disease in the game of publication is bad refereeing, a catchy name for which is solicited to match Impactitis.
